# Diagnosing Hip Microinstability: an international consensus study using the Delphi methodology

**DOI:** 10.1007/s00167-022-06933-4

**Published:** 2022-04-30

**Authors:** Vikas Khanduja, Nicholas Darby, John O’Donnell, Nicolas Bonin, Marc R. Safran, The International Microinstability Expert Panel, A. J. Andrade, Aaron Krych, Ajay Malviya, Allston J Stubbs, Amir Takla, Athanasios Papavasiliou, Bent Lund, Callum McBryde, Danyal Nawabi, Dave Kohlrieser, Etienne L. Belzile, Johan Witt, Karadi Hari Sunil Kumar, Keelan R Enseki, Laura Diamond, Leandro Ejnisman, Marcus Bankes, Matt Wilson, Nicholas Mohtadi, Oliver Marin-Pena, Olufemi Ayeni, Panayiotis Christofilopoulos, Parminder Singh, Richard Field, Soshi Uchida, Sverre Løken

**Affiliations:** 1 Young Adult Hip Service, Addenbrooke’s, Cambridge University Hospital NHS Foundation Trust, Cambridge, UK; 2 St Vincent’s Private Hospital East Melbourne, East Melbourne, Australia; 3 Lyon Ortho Clinic, Lyon, France; 4 Division of Sports Medicine, Department of Orthopaedic Surgery, Team Physician, Stanford University, Redwood City, CA USA

**Keywords:** Microinstability, Delphi, Consensus, Hip pain, Dysplasia

## Abstract

**Purpose:**

Hip microinstability is a relatively new diagnosis which is increasingly being discussed in the literature and yet there are no clear guidelines for making a diagnosis. Microinstability has generally been defined as persistent excessive hip motion that has become symptomatic especially with pain. This aim of this Delphi study was to seek expert opinion to formulate a diagnostic criteria for hip microinstability.

**Methods:**

A Delphi methodology was used for this consensus study. A literature search was conducted on PubMed up to March 2019 using the keywords ((hip) and (microinstability)) to identify relevant articles on this topic. All relevant criteria used for diagnosing hip microinstability were collated to create a questionnaire and further criterion suggested by the experts were included as well. Four rounds of questionnaires were delivered via an online survey platform. Between each round the authors acted as administrating intermediaries, providing the experts with a summary of results and synthesising the next questionnaire. The expert panel was comprised of 27 members: 24 (89%) orthopaedic surgeons and 3 (11%) physiotherapists from around the world.

**Results:**

Expert panel participation in rounds 1–4 was: 27 (100%), 20 (74%), 21 (78%) and 26 (96%) respectively. A literature review by the authors identified 32 diagnostic criteria to populate the first questionnaire. Experts suggested amending three criteria and creating five new criteria. The panel converged on ranking 3 (8%) of criteria as “Not important”, 20 (54%) as “Minor Factors” and 14 (38%) as “Major Factors”. No criteria was ranked as “Essential”. Criteria were subcategorised into patient history, examination and imaging. Experts voted for a minimum requirement of four criteria in each subcategory, including at least six “Major factors”. The final diagnostic tool was approved by 20 (77%) of the final round panel.

**Conclusion:**

This study describes the first known expert consensus on diagnosing hip microinstability. The relative complexity of the final diagnostic tool is illustrative of the difficulty clinicians’ face when making this diagnosis.

**Level of evidence:**

V.

## Introduction

Inherent stability of the hip joint is conferred by the bony anatomy restricting the movement of the femoral head within the acetabulum and the supporting ligaments and capsule [4]. The acetabular morphology confers a basic degree of static stability restricting extreme ranges of motion [43]. Further support is layered onto the osseous architecture comprising the labrum, ligaments, capsule and the muscles of the hip [8]. The labrum provides further stability by deepening the acetabulum and forming a partial seal which creates a negative intra-articular pressure during joint distraction [44]. In cadaveric hips, labral tears have been demonstrated to significantly reduce joint stability [13]. Anterior capsule of the hip joint plays an important role in providing stability to the hip joint [25, 26]. The cadaveric study by Johannsen et al. showed that stretching of the anterior capsule of the hip joint improved rotational movement [25]. In addition, Packer et al. showed that a thin capsule noted on pre-operative MRI was suggestive of laxity in the hip joint intra-operatively [36]. The ischiofemoral, iliofemoral and pubofemoral ligaments each serve to limit specific movements and together form the stabilizing joint capsule [15, 17]. A further ligament, the zona orbicularis wraps around the femoral neck and has been proposed to tighten during joint distraction forming a “locking ring” [22].

Hip instability is characterised by excessive motion, can occur due to a variety of conditions (such as DDH, post-traumatic, connective tissue disorders, microtrauma, idiopathic and iatrogenic) often leading to pain and disability. Hip microinstability is a relatively new diagnosis which has gained increasing recognition both clinically and in the literature over the last decade. Microinstability has generally been defined as persistent excessive hip motion (insufficient to be classed as dislocation or subluxation) that has become symptomatic especially with pain [7, 11, 14, 27, 42]. The condition classically presents in younger patients (16–50 years) which may be explained by increased participation in causative activities or the natural stiffening of joints with age [11, 14, 27, 39]. Females may also be more likely to present with hip microinstability, a theory supported by a demonstration that female ballet dancers show greater joint distraction on split antero-posterior radiographs compared with their male counterparts [14, 32]. In addition the pattern of chondral injury is different in those without dysplasia but having instability in the hip joint [41]. The proposed treatment for hip microinstability is physiotherapy-directed muscle strengthening programme and changes to activity/sport to strengthen the muscles around the hip, abdomen and lower back [7, 14, 27]. Oral anti-inflammatory analgesia may be used to ease symptoms followed by an intra-articular corticosteroid injection (if required after 6–12 weeks of physiotherapy) [7, 27]. In patients who are persistently symptomatic following non-operative treatment with physiotherapy, a surgical approach may be undertaken, either arthroscopically or open, to correct underlying aetiology [7, 14, 27]. Some of the surgical options are periacetabular re-directional osteotomy for acetabular dysplasia, arthroscopic thermal capsulorraphy or plication, thermal shrinkage and labral repair/reconstruction. However iatrogenic microinstability, especially in deep flexion, could be caused due to over resection of cam lesion [23, 33].

Despite an increased interest, hip microinstability remains a challenging diagnosis for clinicians to make. One of the main hurdles clinicians face when diagnosing microinstability is a lack of clear and objective diagnostic criteria [27]. Consequently, current clinical diagnosis must rely predominantly on the individual opinions of experts. Similarly, for studies reported in the literature, microinstability is either defined arbitrarily by researchers setting their own “gold standard” diagnostic criteria or by reproducing the criteria used in previous studies [9, 18, 45]. Currently there is no consensus between experts regarding both the relative importance of criteria and which criteria must be satisfied to make a diagnosis of hip microinstability [24]. This aim of this online Delphi study was to seek the expert opinion to answer the above questions and formulate diagnostic criteria for hip microinstability.

## Materials and methods

### The Delphi technique

The Delphi technique was first designed by the RAND corporation in the 1950’s and since conception the general principles that define this methodology have remained unchanged. The Delphi technique is used to arrive at a consensus on a topic wherein a group of experts are anonymously asked whether they agree or disagree on a particular question/idea. The core features of a Delphi study are as follows: experts answer a questionnaire anonymously, the answers are collected by the study facilitators, the aggregated results are fed back to the experts in a standardised format and then the entire process is repeated for multiple iterations [3, 20, 34, 38]. The experts may work towards agreement or a significant percentage of them might refuse to agree on some aspects which is also important to disregard a criterion.

When compared with the alternative approaches for achieving a group consensus Delphi methodology boasts a number of advantages and has been suggested to outperform face-to-face meetings [38]. The anonymity that underlies a Delphi study removes the notion of direct challenging between individuals. This reduces the tendency to mount a rigid defence of one’s original stance and prevents dominant personalities from exerting a disproportionate influence over the group [34]. Eliminating a face-to-face meeting is logistically convenient and affords experts the time and privacy to consider the aggregated feedback before answering the next questionnaire. The main disadvantages of Delphi studies include expert panel drop out (especially with increasing iterations) and the introduction of facilitator bias [3]. These impacts can be respectively minimalised by limiting the number of iterations, ensuring questionnaires remain relatively short and allowing expert panel members to contribute their own questions [3]. Delphi study methodology has extensive examples of field applications throughout industry, academia and medicine, including orthopaedics [2, 30, 37].

### Study design

It has previously been suggested that a minimum of 12 experts are required for a study such as this one [6]. In this study the expert panel comprised of 27 experts: 24 (89%) orthopaedic surgeons and 3 (11%) physiotherapists. Expert panel selection was done on the basis of years in practice, track record of peer-reviewed publications in the area of hip preservation surgery, experience in the management of hip instability and geographical location to ensure a global representation. A literature search was performed on PubMed up to March 2019 using the keywords ((hip) and (microinstability)) to identify relevant articles published on this topic. There were 45 articles retrieved with the search terms, out of which 25 were selected for full text review after screening the abstracts. Two authors (ND and VKH) reviewed the final selected articles pertaining to hip microinstability and formulated a list of criteria suggested to be relevant for diagnosis. This list was termed the “diagnostic criteria” and divided into 3 subcategories: patient history, examination and imaging. The study consisted of four questionnaires created and distributed between April 2019 and March 2020 using the online survey platform *Survey Monkey® *(*Survey Monkey, San Mateo, California*)*.* In between each round the facilitators collected and presented the answers within a summary document which was circulated to the experts for review prior to the next round.

In the first-round experts ranked the randomly ordered diagnostic criteria as either: not important, minor factor, major factor or essential for making the diagnosis of hip microinstability. A 4-point scale was favoured as this has previously been shown to yield stable responses in previous Delphi studies [1, 46]. Criteria were assigned the most popular (modal) ranking. Experts were asked an open question for any other criteria to be included in this round. In the second-round experts were asked whether they agreed or disagreed with the modal ranking assigned to each criterion. There exists no clear guidance on the consensus cut offs that facilitators should use [20]. A criterion’s ranking was finalised if it received 50% or greater agreement from the expert panel. If a criterion’s ranking received less than 50% agreement, it was re-ranked as the next most popular answer and submitted back to the experts for another vote. In the third-round experts were presented with the diagnostic criteria organised into a tabular format with rankings as row headings and subcategories as column headings. Experts were asked to state the minimum number of criteria patients must fulfil in each row, each column and in total. For example, an expert may respond “at least 3 criteria must be fulfilled in the Major factor row and at least 5 criteria in the Minor factor row, these criteria must be distributed such that there are at least 2 criteria in each of the Patient History, Examination and Imaging columns”. Median numbers were calculated to avoid the averages being skewed by anomalous answers. In the fourth-round experts were shown the final diagnostic tool and asked whether they approved or disapproved it.

**Fig. 1:**
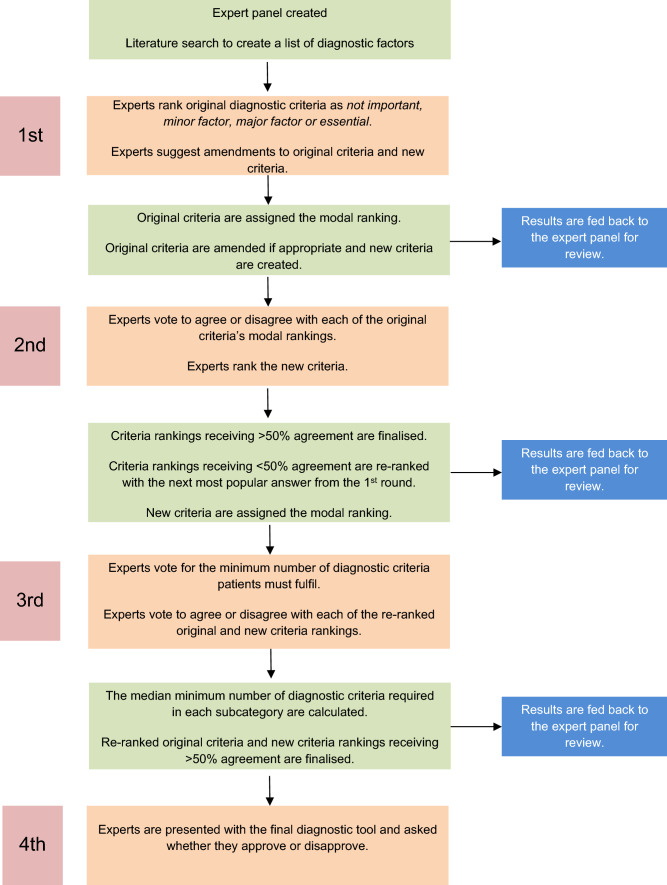
Consensus methodology steps

At the beginning of this study (during the first round) experts were invited to suggest amendments to the diagnostic criteria or suggest their own diagnostic criteria. This was done to reduce the effect that facilitator bias would have on the final outcome [3]. New criteria were fed into the study to be ranked and finalised in the same manner as the original criteria. By necessity of their conception in the first round the new criteria subsequently lagged one round behind the original criteria as shown in Fig. 1.

## Results

Expert panel participation was: 27 (100%), 20 (74%), 21 (78%) and 26 (96%) in rounds 1–4 respectively. The initial literature search by the authors identified 32 diagnostic criteria to populate the first questionnaire.

In the first round 3 (8%) of the criteria received a modal ranking of “Not important”. The most common modal ranking was “Minor factor”, which 20 (54%) of the criteria received. A further 14 (38%) of the criteria received a modal ranking of “Major factor”. No criteria received a modal ranking of “Essential”. The expert panel submitted 10 written suggestions which were reviewed by the facilitators and used to amend 3 existing criteria and create 4 new criteria.

In the second round 31 (97%) of the original criteria rankings received > 50% agreement by the expert panel and were subsequently finalised. Three of the criteria received a finalised ranking of “Not important” and were discarded. The one (3%) criterion ranking that received < 50% agreement was re-ranked from a “Major factor” to a “Minor factor”. Of the 4 new criteria, 2 (50%) received a modal ranking of “Major factor” and 2 (50%) received a modal ranking of “Minor factor”.

In the third round all 5 (100%) of the re-ranked criteria or new criteria rankings received > 50% agreement by the expert panel. Within the patient history the median minimum number of criteria required was 4 (mean = 4, mode = 2, range 1–9). Within the examination the median minimum number of criteria required was 4 (mean = 4, mode = 5, range 1–8). Within the imaging/arthroscopy the median minimum number of criteria required was 4 (mean = 4, mode = 2 and 6, range 1–10). The median minimum number of criteria that must be “Major Factors” was 6 (mean = 6, mode = 3, range 3–11). Finally, the median minimum number of total criteria was 10 (mean = 12, mode = 10, range 4–28), because this was less than the sum of the minimum requirement across history, examination and imaging/arthroscopy this result was obsolete.

In the final round, due to a new and relevant publication, an additional criterion was created: “Does the patient have a FEAR index > 5 degrees?”. The modal ranking received by this criterion was “Major Factor”. The final diagnostic criteria (inclusive of the FEAR index criterion) was approved by 20 (77%) of the fourth-round panel. The final diagnostic criteria for hip microinstability is shown in Table 1.

**Table 1:**
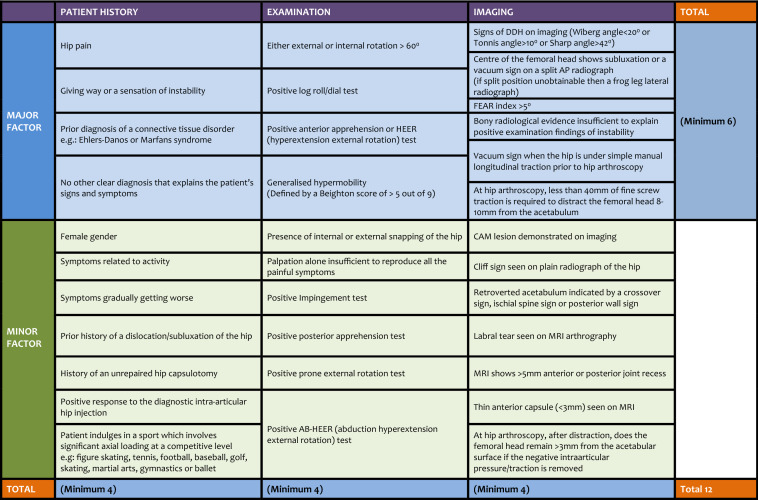
Microinstability diagnostic criteria

## Discussion

Diagnostic criteria for hip microinstability (Table 1) were created following an International Delphi consensus study by hip preservation experts from around the globe. The diagnostic criteria (Table 1) is in tabular format with major and minor factors along the rows and three main columns looking at patient history, examination and imaging. Within these rows and columns are a total of 34 criteria deemed by the expert panel to have diagnostic value. To meet the requirement for diagnosis of microinstability a patient must satisfy the minimum number of criteria required in the major factor row and each of the columns. These minimums, as shown in Table 1, are 6, 4, 4, 4 for the major factor row, patient history column, examination column and imaging column respectively. The creation of this diagnostic criteria aims to address the frequently cited need for consensus and consistency when diagnosing hip microinstability [27].

### Diagnostic features in the patient interview

When diagnosing hip microinstability one of the most important symptoms to explore is persistent pain [7, 11, 12, 18, 19, 27, 39, 42]. This may be reported in the groin, buttock, thigh or in a C-sign distribution. If the patient has previously received an intracapsular hip injection then it is helpful to ask whether the injection improved their symptoms, which would be consistent with an intracapsular pathology [9, 14, 27]. In addition to pain, the patient may report a sensation of instability and giving way, often followed by apprehension [11, 12, 14, 18, 27].

The patient’s past medical history may yield further diagnostic clues. For example an established connective tissue disorder such as Ehlers-Danlos, Marfan’s or Down Syndrome would cause generalised laxity predisposing to hip instability [7, 11, 14, 27]. A history of a previous traumatic injury such as a subluxation or dislocation could represent causative damage to the soft tissue stabilisers of the hip [11, 27]. Alternatively, a surgical history of an unrepaired capsulotomy may represent an iatrogenic origin of microinstability [7, 14, 15, 27].

Finally, the patient’s activities should be explored, with emphasis being placed on axial loading sports such as ballet, tennis, golf and gymnastics. This is because these activities require repeated external rotation of an extended hip, a motion which has been suggested to cause repetitive micro trauma and increased laxity over time [7, 11, 27, 42].

### Diagnostic findings in the patient examination

The intracapsular pain caused by hip microinstability should not normally be reproduced during simple palpation [27]. Several provocative and/or dynamic tests have been suggested to have diagnostic potential. The log roll (or dial) test and posterior apprehension test may be performed to assess anterior and posterior capsular laxity respectively [7, 14, 27]. In addition, the Fitzgerald test may be performed to indicate the presence of a labral tear [16].

Further tests of diagnostic value have previously been evaluated by Hoppe et al., these include: the anterior apprehension test, prone external rotation test and abduction hyperextension external rotation test (AB-HEER) [21]. Hoppe et al. defined microinstability as meeting one of several gold standard criteria which centred around either objective measures of hip distraction or pathological arthroscopic findings [21]. Compared with these gold standard criteria the three specialist tests were reported to have sensitivities ranging from 34 to 81% and specificities ranging from 85 to 98% [21].

Finally, the iliopsoas tendon has been suggested to compensate for laxity by tightening, which may in turn increase friction when moving over the anterior bony architecture of the hip [5, 11]. As a result of this process, examining the patient for signs of coxa saltans (hip snapping) may have diagnostic value [5]. Furthermore, the deep hip muscles are becoming increasingly important in assessing the muscular stability of the joint and perhaps the lack of muscular endurance may suggest a degree of instability.

### Diagnostic signs in the patient imaging and arthroscopy

One of the most frequently cited methods of diagnosing hip microinstability revolves around utilising x-ray or fluoroscopy to visualise the femoral head distracting from the acetabular surface. The choice of force used to demonstrate joint distraction has varied between authors [5, 21, 27, 28, 41]. Once the force has been applied diagnosis is made based on visible separation of the femoral head from the acetabulum, either by direct measurement (> 7–10 mm) or the presence of a vacuum sign on fluoroscopy [27]. The tendency of the joint to remain distracted (> 3 mm) once the traction and negative intra-articular pressure has been removed can provide further confirmation of laxity [21, 27].

Certain osseous abnormalities may cause microinstability and therefore their identification may have diagnostic potential. Quantitative measures of dysplasia include the assessment of the Wiberg, Sharp and Tönnis angles (also known as acetabular index) [14, 29, 40]. A retroverted acetabulum will provide inadequate posterior coverage and such patients may demonstrate an ischial spine or posterior wall sign [14, 27]. In patients with femoroacetabular impingement (FAI) a CAM lesion may lever the femoral head posteriorly [10, 14, 27]. Lastly the Cliff sign (a disruption in the circular continuity of the lateral femoral head) and Femoral Epiphyseal Acetabular Roof index (an angle between the acetabular roof and the central third of the femoral growth plate) are relatively new signs shown to be associated with microinstability [35, 45, 48].

Soft tissue imaging to aid microinstability diagnosis may use MRA to check for a wide anterior joint recess, or a thin capsule (< 3 mm) lateral to the zona orbicularis on an axial oblique image [31]. During arthroscopy the presence of labral tears and the chondral wear pattern should be assessed with particular attention paid to straight anterior tears and an inside out chondral wear pattern, both of which may be associated with instability [26, 27, 41]. More recently Woodward et al. reported that Microinstability was associated with capsular thinning and labral hypertrophy [47].

In this study only 3 criteria were ranked by the expert panel to be “Not important”. These rejected criteria included the patient: being a young adult (18–45 years), having signs of iliotibial band tightness (Ober test) or iliopsoas tendonitis and having ligamentum teres hypertrophy on MRI. Interestingly each of these rejected criteria have been previously citied in the literature in association with hip Microinstability [5, 11, 14, 27].

It is evident when evaluating the final diagnostic criteria produced by this study that the expert panel believe a diagnosis of hip microinstability to be a relatively complex process. With no essential criteria that must be fulfilled, diagnosis is instead based on satisfactory evidence in each of the patient’s history, examination and imaging. This result is surmised in the criterion “Is bony radiological evidence insufficient to explain positive examination findings of instability?”. This criterion implies that, in some cases, the panel believes that process of elimination may play a role in diagnosing hip microinstability.

This study utilised a number of strengths in its Delphi design. Firstly, the expert panel was sufficiently sized and suffered a low dropout rate [6]. Secondly, the study included four rounds allowing experts to re-consider and re-vote on criteria. Finally, experts were invited to suggest new criteria and amend existing ones reducing the impact of facilitator bias.

Delphi methodology was chosen to ensure a global participation of experts who have significant experience in managing hip microinstability. However, there are some weaknesses in this study. Firstly, there is no gold standard on which to base the diagnosis of microinstability, and thus compare the validity of the diagnostic criteria proposed, or the individual tests described. Secondly, there is a potential that the facilitators’ view may have interfered with the analysis with a possibility that differing opinions may not have been fully explored. Thirdly, the FEAR index was introduced in the middle of the study, being a new diagnosis, the literature and scientific exploration and subsequent publications and currently available evidence may be lacking and impacting on achieving the necessary consensus. Fourthly, with the proposed criteria, there is a potential for over diagnosis of hip microinstabiliy. However, as a first step the authors felt that at this stage it was important to include everyone with the listed criteria and then in due course with validatation of the tool further refinement of the criteria would make it robust.

## Conclusion

This Delphi consensus gives a set of criteria to diagnose hip microinstability. This is the first step to standardise the clinical diagnosis as well as provide a suitable reference for research studies which require a “gold standard” for hip microinstability diagnosis. Future work is now required to validate the diagnostic criteria which will allow wider acceptance of this panel’s consensus both amongst academics and clinicians alike.
